# Experimental study and simulations of hydrogen cooling effectiveness for aviation PEM fuel cells

**DOI:** 10.1038/s41598-023-49309-5

**Published:** 2023-12-27

**Authors:** Till Lennart Kösters, Arne Graf von Schweinitz, Michael Heere, Jens Friedrichs, Xin Gao

**Affiliations:** 1 Institute for Internal Combustion Engines and Fuel Cells (ivb), Technische Universität Braunschweig, Braunschweig, Germany; 2 Institute of Energy and Process Systems Engineering (InES), Technische Universität Braunschweig, Braunschweig, Germany; 3 Institute of Flight Propulsion and Turbomachinery (IFAS), Technische Universität Braunschweig, Braunschweig, Germany; 4 SE2A Sustainable and Energy Efficient Aviation, Technische Universität Braunschweig, Braunschweig, Germany

**Keywords:** Fuel cells, Aerospace engineering, Thermodynamics

## Abstract

Proton exchange membrane fuel cells (PEMFCs) are seen as one possible future means of driving the change towards a zero-emission society. In a civil aircraft, fuel cell systems can have multiple potential benefits, such as reduced noise, lowered emissions and higher fuel economy compared to jet aircraft. For controlling the fuel cell temperature, thermal management systems are required which can be optimized for aircraft applications regarding their weight and reliability. In this work, a simplified and light-weight thermal management system relying on hydrogen cooling is presented and analysed. To investigate the feasibility, a test rig and a three-dimensional, singular channel model in ANSYS Fluent were designed. Fuel cell temperature could be maintained within the set threshold in the model and the test rig, thus showing that controlling the fuel cell temperature via the hydrogen reactant flow is a viable alternative thermal management system. Results from the model indicate that both the hydrogen mass flow and hydrogen inlet temperature should be used to control the fuel cell temperature. Furthermore, operating the fuel cell at medium to low current densities is favourable for hydrogen cooling. Future studies will explore alternate flow field designs to facilitate thermal management system relying on hydrogen.

## Introduction

In 2011, the European Commission published the “Flight Path 2050” and drew a set of environmental goals for aviation^1^. Accordingly, until the year 2050, a reduction of CO_2_, NO_X_ and noise emissions shall be achieved by 75%, 90% and 60% respectively. These values refer to a typical aircraft that was manufactured in the year 2000. Considering the increasing amount of air travel, these goals are unlikely to be reached by evolutionary improvements of existing aviation technology^2^. It is widely accepted that using hydrogen fuel cell systems for main propulsion power in a civil aircraft can improve the design in multiple ways, such as reduced noise, lowered emissions and higher fuel economy^3^. This makes fuel cell powered aircraft better suited than traditional aircraft to meet the “Flight Path 2050” environmental goals. To further strengthen their competencies in the aviation sector, the weight reduction and reliability improvement of these aviation fuel cell system designs are of great importance^4^.

Inside an aviation fuel cell system, the cooling subsystem can take up to 50% of the total system weight^5^. This makes efficient heat rejection increasingly important for an aviation fuel cell system, or in general, for any fuel cell system treating light weight as an important feature. As of now, fuel cells applied in aviation are limited power wise to kilowatts either as primary power supplies on small experimental aircraft or as auxiliary power sources for aircraft^5–7^. To the knowledge of the authors there are few publications and solutions that have elaborately addressed the efficient heat rejection for fuel cell systems up to megawatts as a primary power supply for commercial airliners. Kellermann et al.^8^ demonstrated heat transfer rates of several MW under favorable conditions using the complete aircraft surface. Sozer et al.^9^ also investigated the cooling potential of the aircraft surface through Outer Mold Line cooling for three different aircraft concepts and achieved a heat rejection of up to 18 kW. An air heat exchanger guideline for fuel cell powered aircraft together with an analysis of the generated drag was presented by Kožulović^10^. Kösters et al.^11^ compared liquid cooling to phase change cooling for air heat exchanger onboard fuel cell powered commercial aircraft and found a drag reduction of 23% of the phase change cooling heat exchanger. A recent European patent application by Gao and Kösters^12^ presented the idea of hydrogen cooling for fuel cells in aviation, where the hydrogen reactant flow is used both as fuel and coolant, leading to a possible reduction in size and mass of the thermal management system.

Furthermore, system reliability is of great importance in the aviation sector and is often achieved through system redundancy^13^. Currently, a minimum redundancy of 2.25 is required for a fuel cell-powered airliner to pass the certification based on the state-of-the-art fuel cell reliability index in literature^14^. This however increases the total system weight and limits the payload of the aircraft drastically, making them an uncompetitive solution for clean aviation. Hereon, reducing the total number of the system components, i.e., increasing the system simplicity is seen as an effective direction in enhancing the system reliability. Besides, this is accompanied by a weight reduction of the system, leading to an increased power to weight ratio.

In this work, a simplified and thus potentially lightweight thermal management system relying on hydrogen cooling is presented and analyzed regarding its feasibility in a proton-exchange membrane fuel cells (PEMFCs). This is done experimentally and via a numerical model in ANSYS Fluent.

## Experimental set-up

All experiments were performed on a PEM fuel cell short stack from *ZBT GmbH* operated with hydrogen as fuel and oxygen as oxidizer. The stack is water-cooled, consists of six cells and provides electrical power of up to 100 W at 3.6 V. The balance of plant of the fuel cell stack is done by the *SMART2 Fuel Cell Test System* by *WonATech*. The SMART2 test rig controls the flow rate of the fuel and oxidizer gasses, records the current and voltage of the fuel cell and measures the pressure and temperature of the fuel and oxidizer gasses. The given accuracy of the gas supply unit is $\pm 1 \%$ at a repeatability of $\pm 2 \%$ of the full scale. Additionally, the gas streams can be conditioned by heating, humidifying and pressurizing. The fuel cell stack is cooled by the *RT 180* thermostat from *Lauda* through deionized water. The *SMART2* test rig is operated through a graphical user interface where the above-mentioned parameters can be controlled. Thermal imaging was used to monitor the temperature of the system through a FLIR DM285 multimeter. In the used temperature range, the FLIR DM285 thermal imaging sensor (FLIR Lepton microbolometer) had an accuracy of $\pm 5 \,{\text{K}}$.

**Figure 1:**
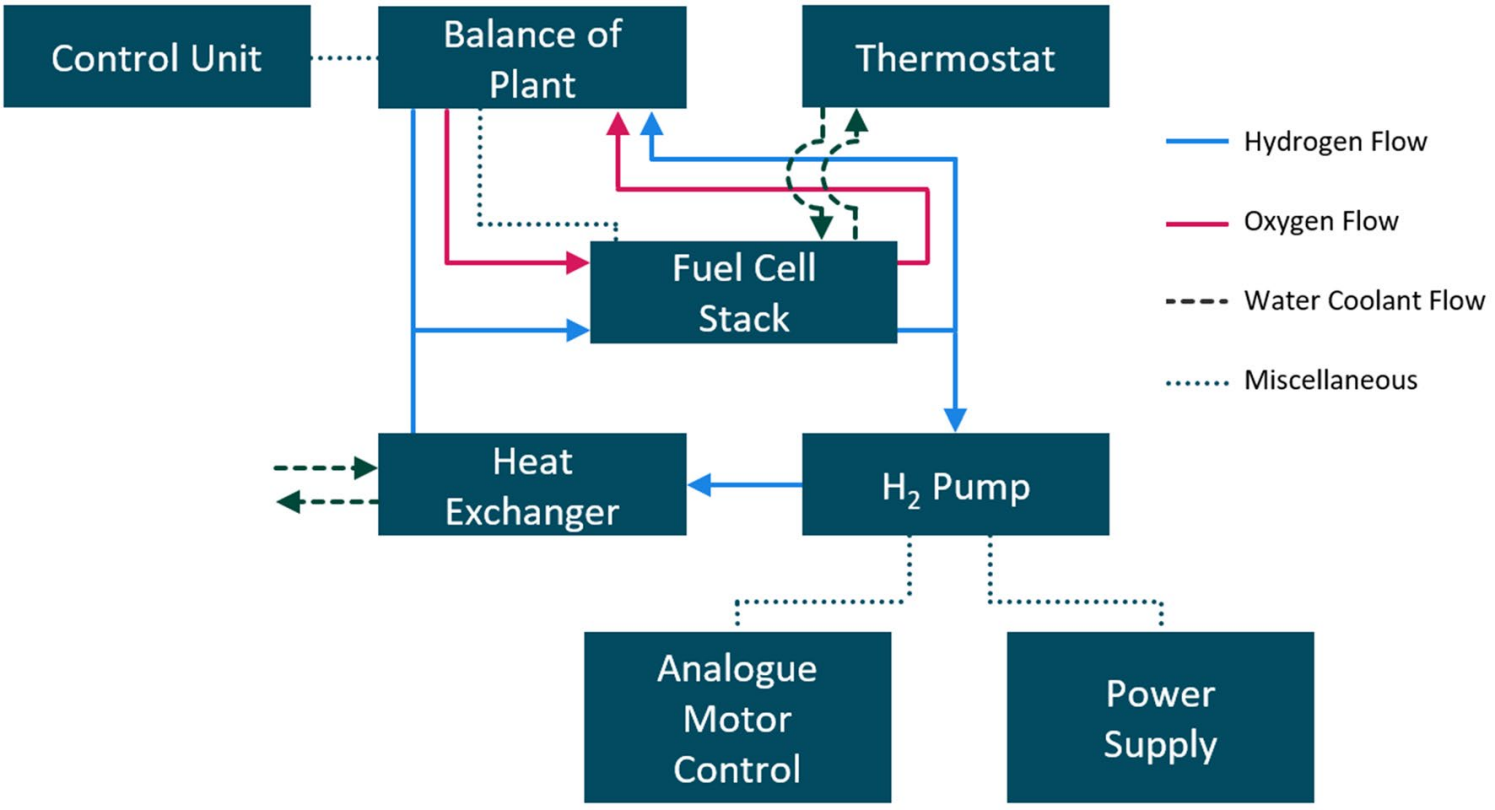
Schematic depiction of the experimental set-up. Main subsystems are marked in dark blue boxes. The media flows are shown in different colors: Hydrogen (light blue), oxygen (red) and water (dark green, dashed line).

The mass flow in the recirculation loop was measured with a *SmartTrek 100* mass flow meter from *Sierra*. The supplier of the mass flow meter states the accuracy as $\pm 1 \%$ and the repeatability as $\pm 0.2 \%$ of the full scale. The hydrogen is recirculated in the loop by the *MH 0018 A* hydrogen recirculation pump from *Busch Vacuum Solutions*. The analogue motor speed control of the hydrogen pump was done by a *VSP 2206* from *Voltcraft*, while the power was supplied by an *EA-PS 3040–40 C* from *EA Elektro Automatik*. The hydrogen was cooled through a plate heat exchanger from *WilTec.* The pipes and fittings to connect the auxiliary equipment were manufactured by *Swagelok*. A schematic representation of the experimental set-up is shown in Fig. 1.

The hydrogen pump was attached to a wooden board which was supported by foam dampeners for electrical insulation. Furthermore, the piping going to and from the pump was realized with perfluoroalkoxy (PFA) tubes. The required electrical insulation by the supplier of ${Z}_{Ohm} \ge 50\, {\text{k}}\Omega$ was achieved with this set-up. The components of the set-up where hydrogen leakage could occur were fitted inside a ventilated space for safety reasons. A casing of foam insulation was constructed for the fuel cell in order to reduce cooling effects from the ambience and the ventilation system.

## Numerical model set-up

In a fuel cell, a wide variety of different physical phenomena occur which are interdependent. In the following, these physical phenomena will be presented with the respective governing equations which are used in the single channel model realized in ANSYS Fluent.

### Electrochemistry modeling

The electrochemistry model adopted in ANSYS Fluent is based on work of Kulikovsky et al., Mazumder and Cole, and Um et al.^15–17^. The main task of the model is to compute the rate of reactions at the anode and cathode. This is done via the surface overpotential, which is the difference between the phase potential of the solid and the phase potential of the ionomer. The resulting electron transport through the solid conductive materials and the protonic transport through the ionomer is modeled as follows.

1 $$\nabla \cdot \left({\kappa }_{sol}\nabla {V}_{sol}\right)+{j}_{sol}=0$$

2 $$\nabla \cdot \left({\kappa }_{ion}\nabla {V}_{ion}\right)+{j}_{ion}=0$$

Here $\kappa$ is the conductivity, $V$ the electric potential and $j$ the volumetric transfer current. The subscripts $ion$ and $sol$ denote the values for the ionomer and the solid respectively. $\nabla$ is the Nabla operator. The proton conductivity of the ionomer ${\kappa }_{ion}$ is modeled according to Springer et al.^18^ as follows.

3 $${\kappa }_{ion}=\left(0.514 {l}_{M}-0.326\right){\text{exp}}\left(1268\left(\frac{1}{303}-\frac{1}{T}\right)\right)$$

where $T$ is the temperature and ${l}_{M}$ the water content of the membrane. For the solid phase, ${j}_{sol}=+ {j}_{cat}$ on the cathode side and ${j}_{sol}=- {j}_{an}$ for the anode side. For the ionomer phase, ${j}_{ion}= -{ j}_{cat}$ on the cathode side and ${j}_{ion}=+ {j}_{an}$ for the anode side. These terms are so-called source terms and are modeled with the simplified Butler-Volmer equation as follows.

4 $${j}_{an}=\left({\upzeta }_{{\text{an}}} {i}_{an}^{ref}\right) {\left(\frac{\left[A\right]}{{\left[A\right]}_{ref}}\right)}^{{\gamma }_{an}}{\cdot e}^{\frac{{a}_{an}F{\eta }_{an}}{\overline{R}T}}$$

5 $${j}_{cat}=\left({\upzeta }_{cat} {i}_{cat}^{ref}\right) {\left(\frac{\left[C\right]}{{\left[C\right]}_{ref}}\right)}^{{\gamma }_{cat}}{\cdot e}^{\frac{{a}_{cat}F{\eta }_{cat}}{\overline{R}T}}$$

Here ${i}^{ref}$ is the reference exchange current density, $\zeta$ the specific active surface area, $\gamma$ the concentration dependence, $a$ the charge transfer coefficient, $\overline{R }$ the universal gas constant and $F$ the Faraday constant. The subscripts $cat$ and $an$ denote the values at the cathode and anode respectively. $[A]$ and $[C]$ represent the molar concentration of the species upon which the reaction rates depend for the anode and cathode respectively. The local surface overpotential $\eta$ quantifies the voltage loss. On the anode side, it can be modeled over the difference between the solid and ionomer potentials. At the cathode side, the open-circuit voltage ${V}_{oc}$ is further subtracted from the difference to account for the gain in electrical potential.

6 $${\eta }_{an}={V}_{sol}-{V}_{ion}$$

7 $${\eta }_{cat}={V}_{sol}-{V}_{ion}-{V}_{oc}$$

For the ionomer phase potential ${V}_{ion}$ there is a zero flux boundary condition as no ionic current leaves the fuel cell through any external boundary. The solid phase potential ${V}_{sol}$ also has zero flux boundary conditions for all external boundaries that are not in contact with the outside electrical circuit. For these contact zones, galvanostatic boundary conditions were chosen.

### Current and mass conservation

The chemical reaction on the anode and cathode side occurs at the so-called triple-phase-boundary of catalyst, ionomer and species. In the triple-phase boundary at the catalysts on the anode and cathode sides, the creation of the different species is modeled as follows.

8 $${S}_{H2}=-\frac{{M}_{H2}}{2 F} {j}_{an}$$

9 $${S}_{O2}=-\frac{{M}_{O2}}{4 F} {j}_{cat}$$

10 $${S}_{H2O}=\frac{{M}_{H2O}}{2 F} {j}_{cat}$$

Here $S$ is the volumetric source term and $M$ the molar mass of hydrogen (${H}_{2}$), oxygen (${O}_{2}$) and water (${H}_{2}O$) respectively. ${S}_{H2}$ and ${S}_{O2}$ are negative as they are being consumed at the triple-phase boundary while ${S}_{H2O}$ is positive as it is being created. As the total electrical current which is generated at the anode and cathode is equal, the current conversation can be stated as follows.

11 $${\int }_{an}{j}_{an}dV={\int }_{cat}{j}_{cat}dV$$

### Heat source

The heat generated in the fuel cell stems from the enthalpy change due to the electrochemical reactions ${h}_{react}$, and the voltage losses which are transformed to heat. The voltage losses can be differentiated into activation losses and ohmic losses. Furthermore, the enthalpy change caused by the condensation and vaporization of water ${h}_{L}$ must be considered. Thus, the heat source term ${S}_{h}$ can be expressed as follows.

12 $${S}_{h}={h}_{react}-{j}_{an,cat} {\eta }_{an,cat}+{I}^{2}{Z}_{ohm}+{h}_{L}$$

Here $I$ is the electrical current and ${Z}_{ohm}$ the ohmic resistivity. The heat transfer coefficient $\alpha$ is calculated based on the combined convective and radiative heat flux $q$, the wall temperature ${T}_{wall}$ and a predefined reference temperature ${T}_{ref}$ as follows.

13 $$\alpha =\frac{q}{{T}_{wall}-{T}_{ref}}$$

### Liquid water formation and transport

The liquid formation and transport model is based on work from^19,20^. In this approach, the conservation equation for the volumetric water saturation $s$ governs the water formation and transport.

14 $$\frac{\partial \left(\Phi {\rho }_{l}s\right)}{\partial t}+\nabla \cdot ({\rho }_{l}{\overrightarrow{u}}_{l}s)={r}_{H2O}$$

Here ${r}_{H2O}$ is the condensation rate for water, $u$ the velocity, $\Phi$ the porosity and $\rho$ the density. The subscript $l$ denotes values for liquid water. The condensation rate is expressed as follows.

15 $${r}_{H2O}={c}_{r}{\text{max}}\left(\left[\left(1-s\right)\frac{{p}_{H2O}-{p}_{s}}{\overline{R}T} {M }_{H2O}\right], \left[-s {\rho }_{l}\right]\right)$$

Here ${c}_{r}$ is the condensation rate constant, ${p}_{H2O}$ the water vapor pressure and ${p}_{s}$ the saturation pressure. For the porous zones, the convective term in Eq. (14) is replaced by a capillary diffusion term.

16 $$\frac{\partial \left(\Phi {\rho }_{l}s\right)}{\partial t} +\nabla \cdot \left[\left({\rho }_{l}\frac{K {s}^{3}}{{\mu }_{l}}\frac{d{p}_{cap}}{ds} \nabla s\right)\right]= {r}_{H2O}$$

Here $t$ is the time, $K$ the absolute permeability, $\mu$ is the viscosity and ${p}_{cap}$ the capillary pressure. The capillary pressure is computed through the Leverett function and thus is dependent on the water saturation and the wetting phase^21^.

17 $$p_{cap} = \left\{ {\begin{array}{*{20}c} {\frac{{{\Gamma }cos\theta }}{{\left( {\frac{K}{{\Phi }}} \right)^{0.5} }} \left( {1.417 \left( {1 - s} \right) - 2.21\left( {1 - s} \right)^{2} + 1.263\left( {1 - s} \right)^{3} } \right)} & {\theta < 90^\circ } \\ {\frac{{{\Gamma }cos\theta }}{{\left( {\frac{K}{{\Phi }}} \right)^{0.5} }} \left( {1.417 \left( {1 - s} \right) - 2.21s^{2} + 1.263s^{3} } \right)} & {\theta > 90^\circ } \\ \end{array} .} \right.$$

Here $\theta$ is the contact angle and $\Gamma$ the surface tension.

The effective diffusivity ${D}_{eff}^{j}$ of the respective gas phase $j$ is modelled via the full multicomponent diffusion method with corrections for the tortuosity of the porous media.

18 $${D}_{eff}^{j}={\Phi }^{1.5} {D}^{ij}$$

Here ${D}^{ij}$ is the binary mass diffusion coefficient which is obtained through the Maxwell–Stefan equations for diffusive mass flux which lead to generalized Fick's law diffusion coefficients^22^.

The model was validated through experimental data from Iranzo et al.^23^. As material and geometric parameters of the ZBT fuel cell stack used in the experiments could not be obtained, the given fuel cell parameters from Iranzo et al. were used for the initial validation of the model. Consequently, the operational parameters were also chosen in compliance with the experimental data from Iranzo et al. An overview of the model parameters for the bipolar plates, gas diffusion layers (GDL), catalyst layers and membrane can be found in Table 1. A mesh sensitivity study was conducted and a mesh of 375,000 elements was chosen to minimize the error while still maintaining an adequate model solving time.

**Table 1:** Model parameters used in the ANSYS Fluent model taken from Iranzo et al.^23^.

Parameter	Value	Units
Bipolar plate density	$1990$	$kg\, {m}^{-3}$
Bipolar plate specific heat capacity	$710$	$J \,k{g}^{-1}{K}^{-1}$
Bipolar plate thermal conductivity	$120$	$W{m}^{-1}{K}^{-1}$
Bipolar plate electric conductivity	$92600$	${\Omega }^{-1}{m}^{-1}$
Bipolar plate thickness	$9.5$	$mm$
Bipolar plate—GDL contact resistance (serpentine)	$3.52\times {10}^{-7}$	$\Omega \,{m}^{2}$
GDL density	$321.5$	$kg\, {m}^{-3}$
GDL porosity	$0.82$	$-$
GDL electric conductivity	$280$	${\Omega }^{-1}{m}^{-1}$
GDL viscous resistance (anode)	$1\times {10}^{12}$	${m}^{-2}$
GDL viscous resistance (cathode)	$3.86\times {10}^{12}$	${m}^{-2}$
GDL wall contact angle	$110$	$deg$
GDL thickness	$420$	$\mu \,m$
Catalyst layer surface-to-volume ratio	$1.25\times {10}^{7}$	${m}^{2} \,{m}^{-3}$
Catalyst layer thickness (anode)	$6.0$	$\mu\, m$
Catalyst layer thickness (cathode)	$12.0$	$\mu \,m$
Catalyst layer porosity	$0.2$	$-$
Membrane density	$1980$	$kg\, {m}^{-3}$
Membrane thermal conductivity	$0.16$	$W\, {m}^{-1}{K}^{-1}$
Membrane equivalent weight	$1100$	$kg \,kmo{l}^{-1}$
Membrane thickness	$175$	$\mu \,m$
Concentration exponent (anode)	$0.5$	$-$
Concentration exponent (cathode)	$1.0$	$-$
Reference exchange current density (anode)	$448\times {10}^{5}$	$\mu \,A \,c{m}_{Pt}^{-2}$
Reference exchange current density (cathode)	$448$	$\mu \,A \,c{m}_{Pt}^{-2}$

**Figure 2:**
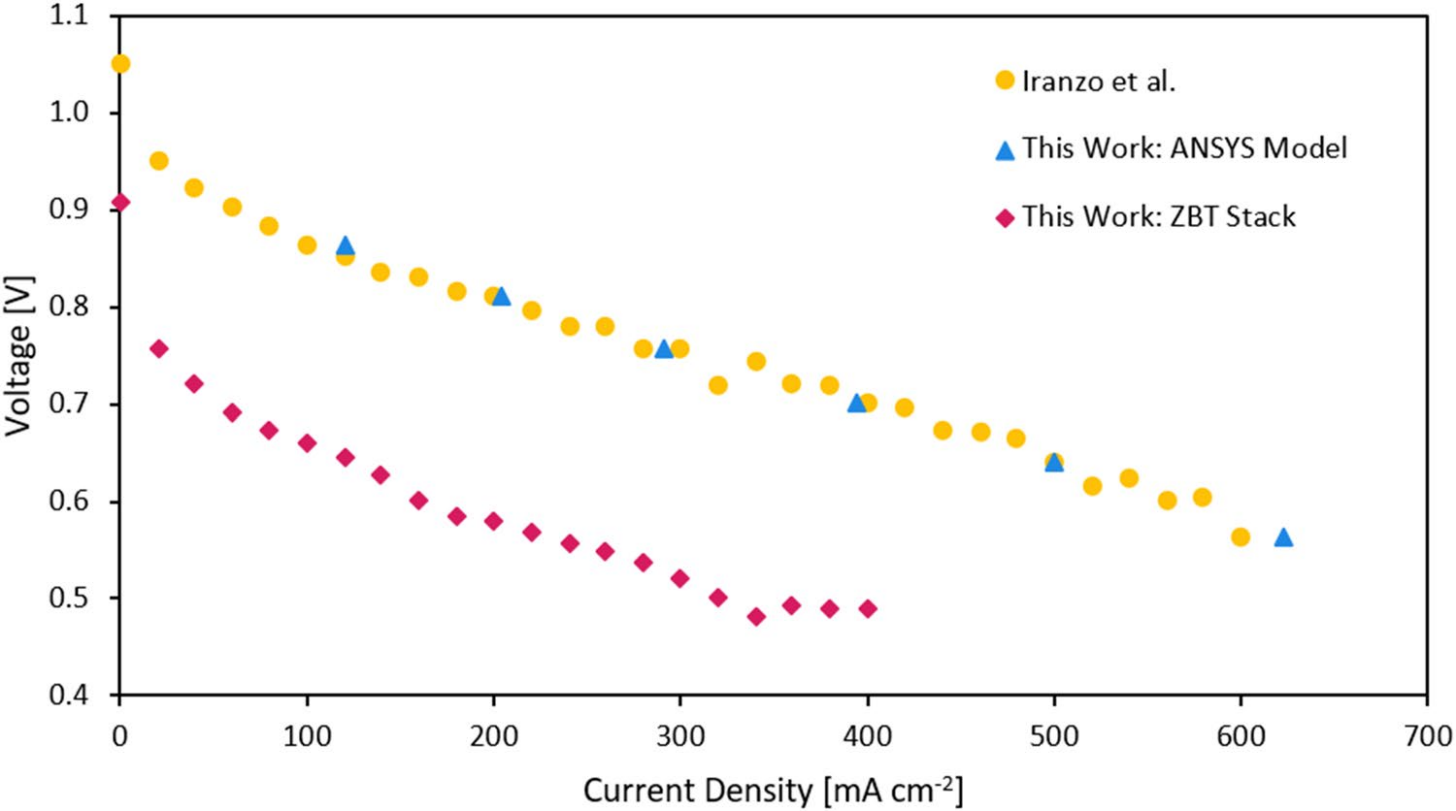
Validation of the ANSYS fuel cell model (light blue) through comparison with experimental data by Iranzo et al.^23^ (yellow). The polarization curve of the ZBT stack is shown in red.

The results of the ANSYS fuel cell model compared to the experimental data of Iranzo et al. can be seen in Fig. 2. The percentage wise error reaches a maximum at the end of the analyzed interval of 6.5%. This is satisfactory, especially since the fuel cell system may primarily be operated at lower current densities ranges which are better suited for aircraft applications^2^. Additionally, the polarization curve of the ZBT stack is shown in Fig. 2 which is used in the experimental part of this study.

## Results and discussion

In the following, the experimental and simulative results will be presented to evaluate the feasibility to achieve sufficient cooling of a PEM fuel cell through the hydrogen reactant stream. First, the overall feasibility will be presented and discussed in the experimental study, followed by an evaluation of beneficial operating parameters through a sensitivity study.

### Experimental study

**Figure 3:**
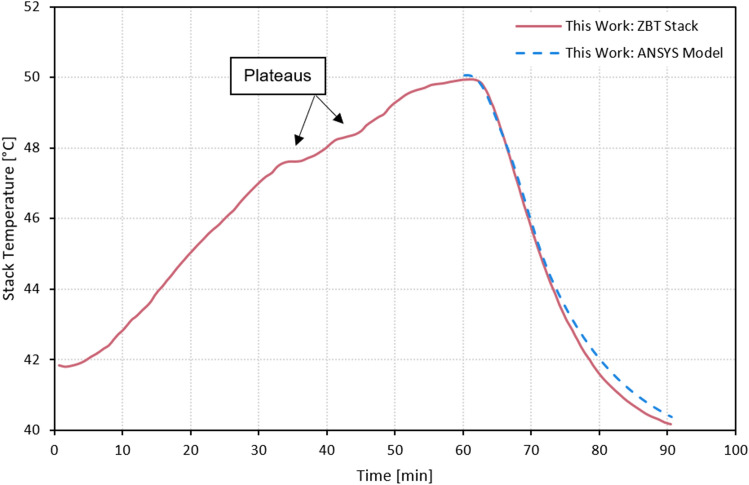
Depiction of the fuel cell stack temperature over time in experiment (red) and simulation (blue, dashed). Hydrogen cooling is initiated after 60 min.

In the following, the results of the parametric, experimental study will be briefly presented. The impact of the hydrogen cooling on the fuel cell stack temperature can be seen in Fig. 3, where experimental and numerical results are combined. The fuel cell was operated at ${I}_{FC}=5 \,{\text{A}}$, ${p}_{op}=2 \,{\text{bar}}$, ${\lambda }_{O2}=2$ and ${\lambda }_{H2}=1.2$ prior to initiation of hydrogen cooling. The inlet gasses were fully humidified. After stopping the initial water coolant flow, the fuel cell temperature rose from ${T}_{FC}=42\,^\circ{\text{C}}$ up to ${T}_{FC}=50\,^\circ{\text{C}}$ over the course of 60 min. It is notable that the fuel cell stack’s temperature did not increase consistently over the 60-min interval. While the reaction kinetics inside the fuel cell are temperature dependent, that does not fully explain the occurring plateaus marked in Fig. 3. It is more likely, that an interaction with other systems such as the residual coolant caused an additional but limited cooling effect. After 60 min, the threshold temperature of ${T}_{FC}=50\,^\circ {\text{C}}$ was reached and hydrogen cooling was initiated. The threshold was chosen so that there is enough buffer to critical membrane temperatures in case hydrogen cooling would not work as anticipated. Initiation of the hydrogen cooling caused a rapid decline of the overall stack temperature and reduces the temperature to ${T}_{FC}=40\,^\circ {\text{C}}$ over an interval of 30 min. In this experimental set-up, the cooling time of the fuel cell was protracted due to the thermal mass of the residual cooling water in the system. The hydrogen pump was operated at 25% load which resulted in a stoichiometric ratio of ${\lambda }_{H2}=65.6$. The hydrogen which did not react within the fuel cell was recirculated by said hydrogen pump back to the hydrogen inlet. It can further be seen that the numerical results accurately follow the experimental temperature curve with the maximum temperature difference being below $\Delta T=0.5 \,{\text{K}}$.

### Numerical model study

In the following, the most representative results of the numerical model study are presented. The entirety of the results of the parametric study can be found in Supplementary Data S1.

#### Variation of hydrogen stoichiometric ratio

**Figure 4:**
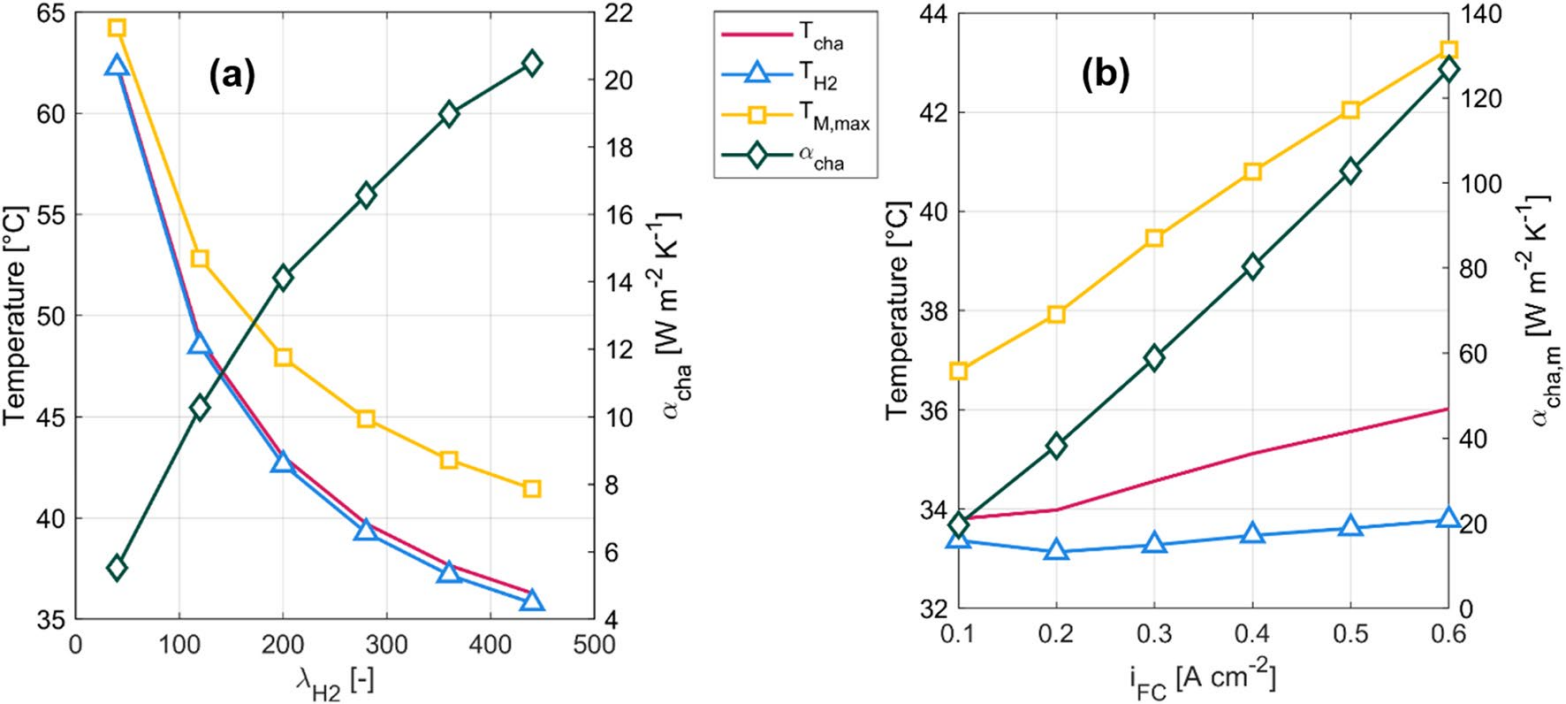
Depiction of the mean channel temperature ${T}_{cha}$ (red), mean hydrogen temperature ${T}_{H2}$ (light blue), maximum membrane temperature ${T}_{M,max}$ (yellow) and mean heat transfer coefficient of the hydrogen channel ${\alpha }_{cha}$ (dark green) for varying hydrogen stoichiometry ratios ${\lambda }_{H2}$ (**a**) and varying current density ${i}_{FC}$ (**b**).

The dependencies of the mean hydrogen channel temperature ${T}_{cha}$, the mean hydrogen temperature ${T}_{H2}$ and the maximum membrane temperature ${T}_{M,max}$ as well as the mean heat transfer coefficient of the hydrogen channel ${\alpha }_{cha}$ to the hydrogen inlet stoichiometry ratio can be seen in Fig. 4a. All temperature values, especially the maximum membrane temperature, are throughout the chosen interval within the operating temperature regions of common PEM fuel cells. Furthermore, the curves of all three temperatures decrease over the interval and flattens out for greater hydrogen stoichiometry ratios as it approaches the inlet hydrogen flow temperature. The mean heat transfer coefficient of the hydrogen channel follows an opposite trend and shows to be sensitive to the hydrogen stoichiometry ratio. The difference between the mean hydrogen temperature and the maximum membrane temperature increases over the interval from $\Delta T=2.02 \,{\text{K}}$ up to $\Delta T=5.66 {\text{K}}$.

**Figure 5:**
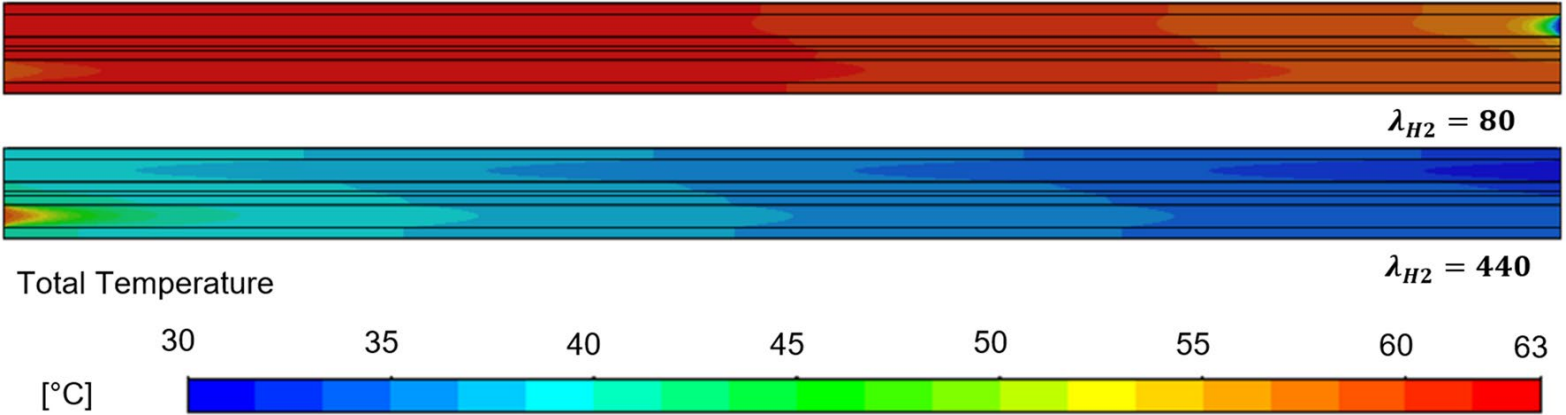
Local distribution of the temperature along the fuel cell channel geometry for ${\lambda }_{H2}=80$ (above) and ${\lambda }_{H2}=440$ (below) at 0.4 $A\, c{m}^{-2}$. Hydrogen enters the channel at the right upper corner.

The local distribution of the temperature along the fuel cell channel can be seen for ${\lambda }_{H2}=80$ and ${\lambda }_{H2}=440$ in Fig. 5. The hydrogen flow enters at the upper right corner while the air flow enters at the lower-left corner. It can be seen that the temperature along the fuel cell channel varies strongly with varying hydrogen stoichiometry ratios and is more uniformly distributed for ${\lambda }_{H2}=80$. This can be quantified by the standard deviation of the mean channel temperature which is minimal for ${\lambda }_{H2}=80$ with ${\sigma }_{Tcha}=1.66 \,{\text{K}}$ and reaches its maximum at ${\lambda }_{H2}=200$ with ${\sigma }_{Tcha}=2.56 \,{\text{K}}$ and decreases down to ${\sigma }_{Tcha}=2.08 \,{\text{K}}$ for ${\lambda }_{H2}=440$.

**Figure 6:**
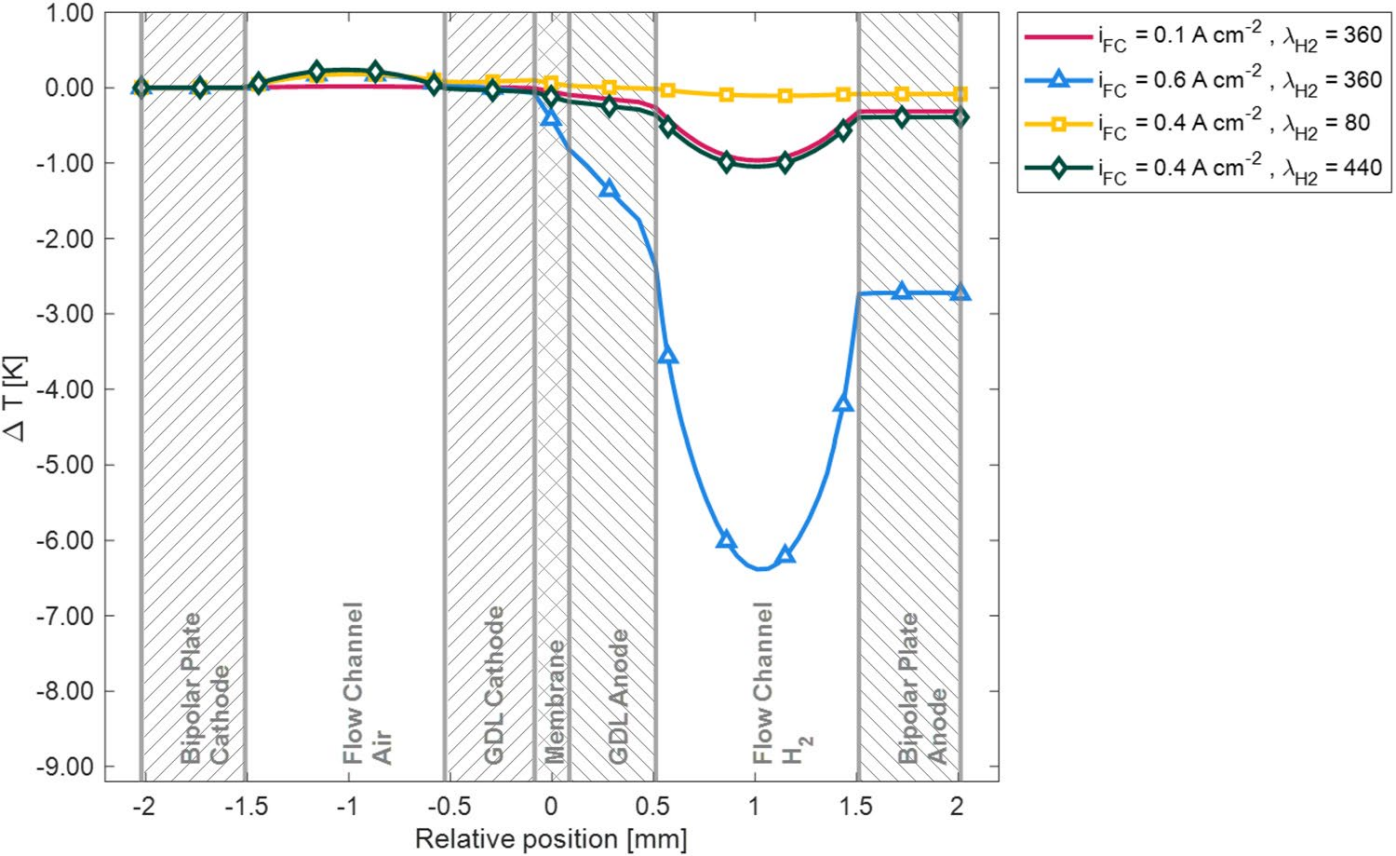
Comparison of the local temperature difference distribution across the cell at half channel length. Red: ${i}_{FC}=0.1 \,{\text{A cm}}^{-2}$ at ${\lambda }_{H2}= 360$, light blue: ${i}_{FC}=0.6 \,{\text{A cm}}^{-2}$ at ${\lambda }_{H2}= 360$, yellow: ${i}_{FC}= 0.4 \,{\text{A cm}}^{-2}$ at ${\lambda }_{H2}= 80$, dark green: ${i}_{FC}= 0.4 \,{\text{A cm}}^{-2}$ at ${\lambda }_{H2}= 440$. For visibility reasons, only a subset of the data is highlighted as markers.

A comparison of the temperature distributions for ${\lambda }_{H2}=80$ and ${\lambda }_{H2}=440$ across the channel at half-length can be seen in Fig. 6. For better comparison, the temperature difference based on the relative temperature of the cathode bipolar plate was plotted. The regions of the fuel cell geometry are marked in this frontal view of the geometry. It can be seen that for ${\lambda }_{H2}=440$ the overall temperature difference between the anode and cathode bipolar plates is more pronounced. Furthermore, the temperature gradient across the channel and the gas diffusion layers is also higher. The maximum temperature in both cases is reached in the cathode channel.

The required blower power is exceptionally sensible to changes of the stoichiometry. The parasitic power consumption of the blower can be displayed as percentage respective to the power produced by the fuel cell channel. For a stoichiometry of ${\lambda }_{H2}=40$ the relative parasitic power amounts to 0.006% but increases to 0.628% for ${\lambda }_{H2}=440$.

#### Variation of hydrogen inlet temperature

**Figure 7:**
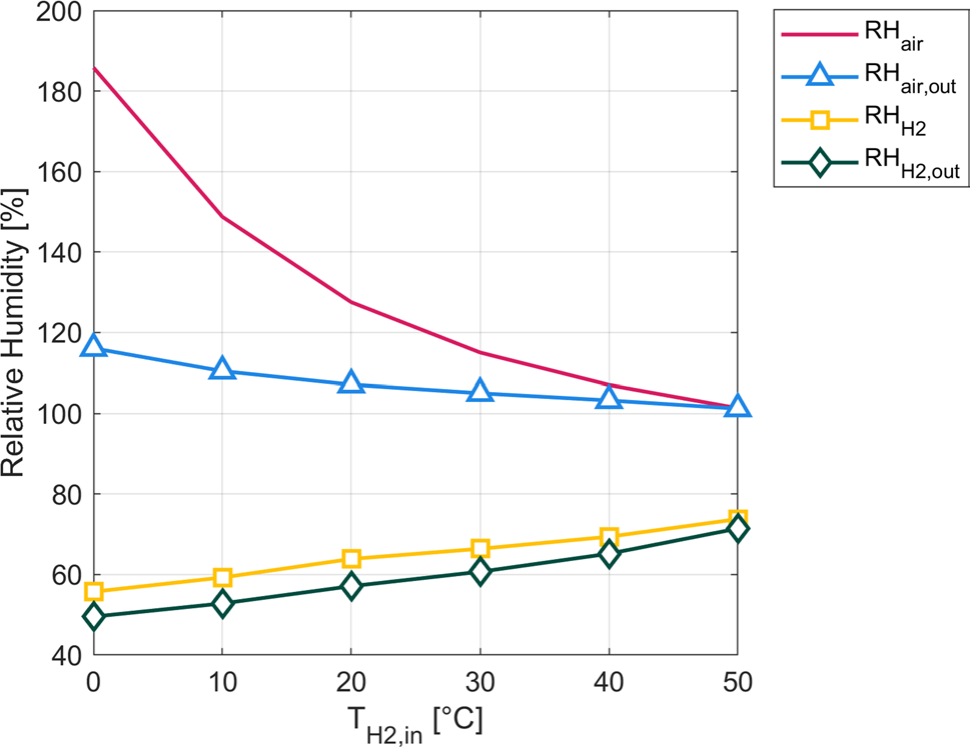
Depiction of the mean air relative humidity $R{H}_{air}$ (red), air relative humidity at outlet $R{H}_{air,out}$ (light blue), mean hydrogen relative humidity $R{H}_{H2}$ (yellow) and hydrogen relative humidity at outlet $R{H}_{H2,out}$ (dark green) as a function of the hydrogen inlet temperature ${T}_{H2,in}$.

In Fig. 7, a representation of the mean air relative humidity $R{H}_{air}$, air relative humidity at outlet $R{H}_{air,out}$, mean hydrogen relative humidity $R{H}_{H2}$ and hydrogen relative humidity at outlet $R{H}_{H2,out}$ as a function of the hydrogen inlet temperature ${T}_{H2,in}$ is presented. It is notable, that the mean hydrogen relative humidity and the outlet hydrogen relative humidity follow approximately the same upward trend over the chosen interval. Simultaneously, the mean air relative humidity as well as the air outlet relative humidity decrease with increasing values of ${T}_{H2,in}$. Especially the mean air relative humidity is relatively high for lower values of the hydrogen inlet temperature with a maximum of $R{H}_{air}=185 \%$ for ${T}_{H2,in}=273.25 \,{\text{K}}$. It then decreases sharply until being equal to the air outlet relative humidity at around $R{H}_{air}=101 \%$ for ${T}_{H2,in}=323.15 \,{\text{K}}$. This trend can be explained by the cooling effect of the hydrogen on the air flow as a decrease in the air temperature causes the relative humidity to rise. The minimum water content of the membrane ${l}_{M,min}$ follows the same upward trend of the hydrogen relative humidity by increasing from ${l}_{M,min}= 6.7 \,{\text{mol {H}}}_{2}{\text{O}} {\left({\text{mol S{O}}}_{3}\right)}^{-1}$ to ${l}_{M,min}= 7.9  \,{\text{mol {H}}}_{2}{\text{O}} {\left({\text{mol S{O}}}_{3}\right)}^{-1}$ over the analyzed interval, showing overall better humidification for higher hydrogen temperatures.

#### Variation of fuel cell current density

In Fig. 4b the mean channel temperature ${T}_{cha}$, the mean hydrogen temperature ${T}_{H2}$, the maximum membrane temperature ${T}_{M,max}$ and the mean heat transfer coefficient of the hydrogen channel ${\alpha }_{cha}$ as a function of the current density ${i}_{FC}$ can be seen. With increasing current density, the maximum temperature of the membrane increases up to a maximum of ${T}_{M,max}=316.4 \,{\text{K}}$ for ${i}_{FC}=0.6 \,{\text{A c{m}}}^{-2}$. This can be explained by the rising heat production due to the increase in generated power and decrease in fuel cell efficiency. Consequently, the mean hydrogen temperature and mean hydrogen channel temperature increase over the set interval and reach at ${i}_{FC}=0.6 \,{\text{A c{m}}}^{-2}$ maximum values of ${T}_{cha}=309.2 \,{\text{K}}$ and ${T}_{H2}=306.9 \,{\text{K}}$.

The initial decrease of the mean hydrogen temperature might stem from the increased hydrogen mass flow as fuel consumption rises due to increased current density and the hydrogen stoichiometry ratio ${\lambda }_{H2}=360$ was kept constant. The stoichiometric hydrogen factor was chosen based on preliminary simulations, as a higher heat production for greater current densities was anticipated. For greater current densities, the increasing generated heat seems to surpass the increase in cooling capacity due to a higher hydrogen mass flow, leading to an increase in overall temperature. This explanation is supported by the steady increase of the mean heat transfer coefficient ${\alpha }_{cha}$ over the set interval as the heat transfer is facilitated due to the higher mass stream and the increased temperature difference between the hydrogen flow and the membrane.

The temperature distribution across the channel at medium length for current densities of ${i}_{FC}=0.1 \,{\text{A c{m}}}^{-2}$ and ${i}_{FC}=0.6 \,{\text{A c{m}}}^{-2}$ is shown in Fig. 6. It is visible that for ${i}_{FC}=0.1 \,{\text{A c{m}}}^{-2}$ the temperature difference across the channel is with $\Delta T=0.98 \,{\text{K}}$ below one Kelvin, whereas for ${i}_{FC}=0.6 \,{\text{A c{m}}}^{-2}$ the temperature difference totals $\Delta T=6.57 \,{\text{K}}$. It has to be noted though that this maximum temperature difference is reached when comparing the hot cathode bipolar plate to the lowest temperature in the hydrogen flow. When it comes to safety concerns however, the maximum temperature difference of the fuel cell components is of greater importance. As can be seen, the maximum temperature difference of the fuel cell components is between the cathode and anode bipolar plates. The temperature difference of the bipolar plates is $\Delta T=0.32 \,{\text{K}}$ and $\Delta T=2.72 \,{\text{K}}$ for ${i}_{FC}=0.1 \,{\text{A c{m}}}^{-2}$ and ${i}_{FC}=0.6 \,{\text{A c{m}}}^{-2}$ respectively. This increase in temperature difference for higher current densities can be attributed to poor thermal conductivity in the MEA and GDL of the fuel cell.

#### Variation of fuel cell channel geometry

Besides the straight channel geometry presented above, two additional fuel cell channel geometries were studied to quantify the pressure drop along the geometry. A L- and U-shape were chosen to reflect critical geometric aspects of a parallel and serpentine flow field respectively. The three geometries were evaluated for ${i}_{FC}=0.4 \,{\text{A c{m}}}^{-2}$, ${\lambda }_{H2}=280$ ${p}_{op}= 4 \,{\text{bar}}$, ${T}_{H2,in}=30\,^\circ {\text{C}}$ and fully humidified inlet gasses. The pressure gradient $\Delta \,p$ over the channel for the three studied geometries I-, L-, U-shape for identical operation parameters and channel length was found to be $43.49 \,{\text{Pa}}$, $47.69 \,{\text{Pa}}$ and $54.68\,{\text{Pa}}$ respectively. The pressure gradient increased from the I-shape to the L-shape by 9.66% and for the I-shape to the U-shape by 25.73%.

The results indicate that a parallel flow field is advantageous for hydrogen cooling as the pressure drop is significantly reduced. Furthermore, the mean channel length of a parallel flow field is smaller than the mean channel length of a serpentine flow field which facilitates the hydrogen cooling as temperature gradients along the respective channel are reduced. Common flow fields are not designed for the high stoichiometry ratios required for hydrogen cooling. Thus, the efficiency of hydrogen cooling can most likely be increased by adapting and optimizing the flow field.

## Conclusions and outlook

In order to investigate the feasibility of a solely hydrogen cooled proton-exchange membrane fuel cell, a test rig was constructed and a three-dimensional, singular channel fuel cell model was created in ANSYS Fluent. The model was used to perform a parametric study of the operational parameters to find a potential optimal operation point.

Adequate cooling of the fuel cell was possible while staying within the threshold of the defined safety parameters. The results indicate that varying the stoichiometry ratio of the hydrogen flow and the hydrogen inlet temperature is well suited for controlling the fuel cell temperature. However, especially for longer channel geometries, these two parameters must be handled with great care as both come with respective challenges. On the one hand, high stoichiometry lead to a fast increase of the necessary blower power. On the other hand, changing the inlet temperature of the hydrogen to the lower regions of the interval leads to undesired high-temperature gradients along the channel. This effect might be less pronounced when analyzing a complete flow field instead of a singular straight channel. Furthermore, low inlet hydrogen temperatures require an oversaturated hydrogen flow to prevent dry conditions. This might prove harmful however when analyzing a complete flow field as channels may be flooded.

Although cooling of the fuel cell model via hydrogen cooling was feasible for all chosen current densities, care must be taken when choosing the operating point. As more heat is produced for greater current densities, the cooling of the fuel cell also becomes more challenging. Depending on the application of the fuel cell, lower current densities might be favorable to minimize the overall temperature gradient and the blower power. In addition, it was shown that the thermal conductivity of the fuel cell MEA may be a limiting factor, as the increased temperature gradient across the channel can be largely attributed to poor thermal conductivity of the MEA components. This effect could be drastically reduced by using MEA materials with better thermal conductivity. Moreover, this would also enhance the cooling capabilities of other thermal management systems, as this problem is inherent to the fuel cell system. It is anticipated that a parallel flow field is better suited for hydrogen cooling due to reduced pressure loss and shorter mean channel lengths.

The feasibility of hydrogen cooling was also shown in experiments on the designed test rig for operation at low current densities. After initiating hydrogen cooling, the stack temperature converged rapidly from 50 °C to 40 °C for a hydrogen stoichiometric factor of ${\lambda }_{H2}=65.6$. This behavior could be replicated in the created numerical model. The results show the viability of hydrogen cooling as an alternative, potentially light-weight thermal management system.

Future studies will explore the advantages of alternate flow field design to facilitate hydrogen cooling. Conducting a variation of material parameters regarding the fuel cell stack is also aspired to improve the efficiency of hydrogen cooling for fuel cells.

### Supplementary Information

Supplementary Information.

## Data Availability

The data generated or analysed during the parametric study are included in this published article and its supplementary information files. The data from the CFD analysis can be accessed from the corresponding author X. Gao upon reasonable request.
